# Predicting patient outcomes in psychiatric hospitals with routine data: a machine learning approach

**DOI:** 10.1186/s12911-020-1042-2

**Published:** 2020-02-06

**Authors:** J. Wolff, A. Gary, D. Jung, C. Normann, K. Kaier, H. Binder, K. Domschke, A. Klimke, M. Franz

**Affiliations:** 1 Department of Psychiatry and Psychotherapy, Medical Center - University of Freiburg, Faculty of Medicine, University of Freiburg, Freiburg, Germany; 2 Department of Business Development, Evangelical Foundation Neuerkerode, Braunschweig, Germany; 3 Department of Business Development, Forensic Commitment and Quality Management, Vitos GmbH, Kassel, Germany; 4 Vitos Hospital for Psychiatry und Psychotherapy, Kassel, Germany; 5 Institute of Medical Biometry and Statistics, Medical Center - University of Freiburg, Faculty of Medicine, University of Freiburg, Breisgau, Germany; 6 Vitos Hochtaunus, Friedrichsdorf, Germany; 7 Heinrich-Heine-University, Düsseldorf, Germany; 8 Vitos Hospital Giessen-Marburg, Giessen, Germany; 9 Justus-Liebig-University, Giessen, Germany

**Keywords:** Psychiatry, Hospitals, Decision support techniques, Machine learning, Health services administration

## Abstract

**Background:**

A common problem in machine learning applications is availability of data at the point of decision making. The aim of the present study was to use routine data readily available at admission to predict aspects relevant to the organization of psychiatric hospital care. A further aim was to compare the results of a machine learning approach with those obtained through a traditional method and those obtained through a naive baseline classifier.

**Methods:**

The study included consecutively discharged patients between 1st of January 2017 and 31st of December 2018 from nine psychiatric hospitals in Hesse, Germany. We compared the predictive performance achieved by stochastic gradient boosting (GBM) with multiple logistic regression and a naive baseline classifier. We tested the performance of our final models on unseen patients from another calendar year and from different hospitals.

**Results:**

The study included 45,388 inpatient episodes. The models’ performance, as measured by the area under the Receiver Operating Characteristic curve, varied strongly between the predicted outcomes, with relatively high performance in the prediction of coercive treatment (area under the curve: 0.83) and 1:1 observations (0.80) and relatively poor performance in the prediction of short length of stay (0.69) and non-response to treatment (0.65). The GBM performed slightly better than logistic regression. Both approaches were substantially better than a naive prediction based solely on basic diagnostic grouping.

**Conclusion:**

The present study has shown that administrative routine data can be used to predict aspects relevant to the organisation of psychiatric hospital care. Future research should investigate the predictive performance that is necessary to provide effective assistance in clinical practice for the benefit of both staff and patients.

## Introduction

The individual needs of patients are central to decision making in hospital care [1]. Nevertheless, reducing complexity of individual episodes through the identification of common patterns of needs facilitates an efficient organisation of care [2].

The identification of common patterns of needs and the prediction of relevant aspects of patient care were found to be more complex in hospital psychiatry than in other medical disciplines [3–5]. Reasons put forward for this were less distinct diagnostic concepts [6–8], less standardisation of care [9] and a broader spectrum of acceptable therapeutic regimes [10].

Machine learning is a potent approach to identify and quantify multidimensional patterns in patient and hospital service data [11]. It has gained increasing attention in health care by achieving impressive results, for instance, in early prediction and diagnosis of breast cancer [12], acute kidney injury [13], skin cancer [14], prostate cancer [15], diabetic retinopathy [16] and depression [17]. Other studies applied machine learning to aspects relevant to the organisation of hospital care, such as predicting patient volume in emergency departments [18–20], the management of acute sepsis [21–23] and the daily costs per psychiatric inpatient [24].

The actual use of machine learning applications in routine clinical care often lags behind prominent achievements in research projects. Most published clinical prediction models are never used in clinical practice [25]. A common problem is the availability of useful data at the point of decision making [26, 27].

Previous research has often included a broad set of medical, psychometric and sociodemographic variables of which many should usually not be available at admission of patients at many hospitals [3, 28]. High administrative workload in clinical staff and overall time constraints are prevalent in many health care systems [29]. Therefore, required feature variables should be routinely available at the point of decision making without further curation.

There is currently a lack of evidence informing the performance and usefulness of machine learning applications based on routine data [30]. Our study addresses this lack of evidence by restricting predictive modelling to a set of routinely available feature variables.

The aim of the present study was to use routine data readily available at admission to predict aspects relevant to the organization of psychiatric hospital care. A further aim was to compare the results of a machine learning approach with those obtained through a traditional method and those obtained through a naive baseline classifier.

## Methods

The present study included all inpatient episodes that were admitted to one of nine psychiatric hospitals in Hesse, Germany, and that were discharged between 1st of January 2017 and 31st of December 2018. An inpatient episode was defined as a patient’s stay at the hospital between admission and formal discharge. We excluded patients that were not in the billing class of adult psychiatry of the German lump-sum payment system for psychiatric hospital care, such as child and adolescent psychiatry and patients with mainly psychosomatic ailments. Missing data in outcome variables was addressed with listwise deletion. The study was approved by the ethics commission of the Medical Council Hesse, record number FF116/2017.

Three different modelling approaches were compared: The chosen machine learning approach was a stochastic gradient boosting algorithm implemented in the CARET package in R based on the gradient boosting machine (GBM) by Friedman [31–33], The traditional method was logistic regression with the full set of feature variables used in the machine learning approach. The naive baseline classifier was obtained by using only basic diagnostic groups in a logistic regression. The basic diagnostic groups were F0/G3 Organic mental disorders, F1 Substance-related mental disorders, F2 Schizophrenia, schizotypal and delusional disorders, F3 Affective Disorders and Others.

The required data were obtained from routinely documented information in the electronic medical records and patient administration databases. A restricted set of feature variables was used that should be available in most hospitals at admission of patients. These were 1. the one-dimensional Global Assessment of Functioning Scale (GAF) [34], 2. age, 3. gender, 4. mode and time of admission and 5. a basic diagnostic grouping (F0/G3 Organic mental disorders, F1 Substance-related mental disorders, F2 Schizophrenia, schizotypal and delusional disorders, F3 Affective Disorders and Others).

We used these features to predict the probability of 1. non-response to therapy as defined by failing to reach the next ten-point-interval of the GAF-scale at discharge (e.g. from 21 to 28 was considered as non-response and from 28 to 31 was considered as response), 2. the need for coercive treatment, 3. the need for 1:1 observation, 4. the need for crisis intervention, 5. long length of stay (LOS) above the 85th percentile and 6. short LOS below the 15th percentile.

We divided data into a training set, i.e. patients discharged in the first three quarters of 2017, a validation set, i.e. patients discharged in the last quarter of 2017 and a test set, i.e. patients discharged in 2018. We engineered features and tuned hyperparameters on the basis of the trained models’ performance in the validation data set. The continuous features, i.e. the GAF sore at admission and patients’ age at admission, were standardised to a mean of zero and a standard deviation of one by subtracting the mean of respective variables from each value and dividing the results by the respective standard deviation. The hyperparameter tuning was carried out using the built-in tuning process in the Caret package, modifying each of the four tuning parameters, i.e. boosting iterations, max depth of trees, shrinkage and minimal terminal node size, until a maximum performance was reached in the validation sample. The performance of the final models was assessed in the held-out test data (patients discharged in 2018) to assess performance in future episodes. Thereby, we had trained nine different models, each holding-out one study-site, and used these models to predict the outcomes of patients from the held-out study site to restrict assessment of performance to hospitals not involved in the training process.

We used the area under the Receiver Operating Characteristic curve (ROC) and Precision and Recall plots (PR-Plots) to compare predictive performance. We calculated 95% DeLong confidence intervals for the area under the ROC [35]. Furthermore, we defined different cut-off values for the operationalisation of the models that maximised sensitivity at a minimum precision of 0.2, 0.25 and 0.33, representing 4, 3, and 2 false positives for each true positive prediction, respectively. We chose a threshold of 0.2 to be the minimum for a clinically meaningful application based on previous work of Tomašev et al. [13]. Furthermore, we defined a sensitivity of 0.2 as the minimum threshold for clinically meaningful application.

## Results

The study included 45.388 inpatient episodes. After addressing missing data in outcome variables with listwise deletion, 40.614 episodes were included in further analyses (89.5%). There were no missing data in feature variables after this step. Table 1 shows the characteristics of included episodes.

**Table 1:** Characteristics of inpatient episodes

	2017		2018	
Number of Episodes (n)	20,283		20,331	
Age (years, mean & SD)	48	19	48	19
Female (n & %)	8872	44	8869	44
GAF Admission (mean & SD)	35	12	35	12
Length of Stay (days, median & IQR)	16	8–29	16	8–29
Basic Diagnostic Grouping (n & %)				
F0/G3	2044	10.1	2099	10.3
F1	7485	36.9	7649	37.6
F2	2929	14.4	3047	15.0
F3	5566	27.4	5365	26.4
Others	2259	11.1	2171	10.7
Study site (n & %)				
Site 1	3564	17.6	3716	18.3
Site 2	1313	6.5	1502	7.4
Site 3	2436	12.0	2548	12.5
Site 4	2115	10.4	1983	9.8
Site 5	2159	10.6	2284	11.2
Site 6	3854	19.0	3656	18.0
Site 7	1493	7.4	1446	7.1
Site 8	1636	8.1	1662	8.2
Site 9	1713	8.4	1534	7.5
1:1 Observation (n & %)	265	1.3	265	1.3
Crisis Intervention (n & %)	219	1.1	192	0.9
Non-Response (n & %)	5108	25.2	4617	22.7
Coercive Treatment (n & %)	1306	6.9	1382	6.8

*SD* Standard deviation, *GAF* Global Assessment of Functioning, *IQR* Interquartile range, F0/G3 Organic mental disorders, F1 Substance-related mental disorders, F2 Schizophrenia, schizotypal and delusional disorders, F3 Affective Disorders

**Fig. 1:**
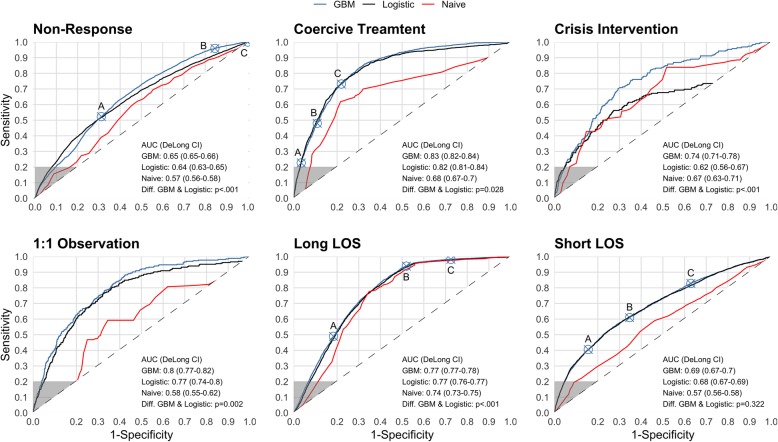
Receiver Operating Characteristic Curves, A = Precision at least 33%, B = Precision at least 25%, C=Precision at least 20%, CI = 95% Confidence Interval. Crossed circles show cut-off values that maximise sensitivity at different minimum thresholds of precision. Grey areas are not clinically meaningful because of a sensitivity of less than 0.2. Cut-off points in grey areas are not shown

Figure 1 compares the possible combinations of sensitivity, i.e. the proportion of correctly predicted actual positives, and specificity, i.e. the proportion of correctly predicted actual negatives, that were reached by the different classifications. The area under the curve is provided for each outcome and classification and 95% confidence intervals were estimated. Furthermore, Fig. 1 shows the operational points at the curves that maximize sensitivity at a minimum precision of 0.2, 0.25 and 0.33, respectively. Measured by the area under the curve, the models for coercive treatment, 1:1 observation, long LOS and crisis intervention achieved a relatively good performance between 0.83 and 0.74.

**Fig. 2:**
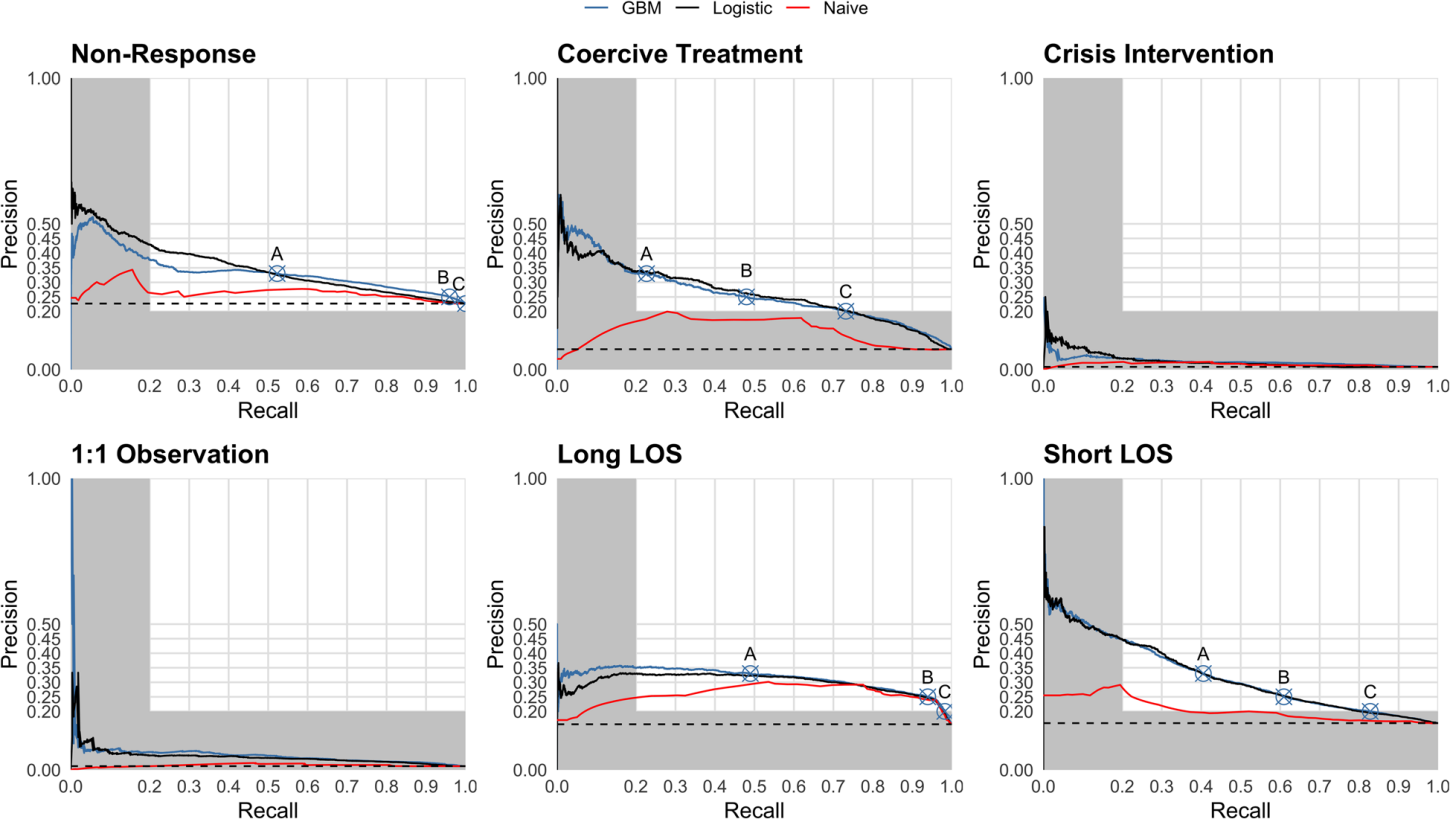
Precision and Recall Plot, A = Precision at least 33%, B = Precision at least 25%, C=Precision at least 20%. Dashed horizontal line shows the prevalence of the outcome. Crossed circles show cut-off values that maximise sensitivity at different minimum thresholds of precision. Grey areas are not clinically meaningful because of a precision or recall of less than 0.2. Cut-off points in grey areas are not shown. Actual precision could be more than minimum precision

Figure 2 compares the possible combinations of recall, a synonym for sensitivity, and precision, i.e. the proportion of actual positives among all positive predictions. Despite a relatively high area under the curve in Fig. 1, the models for the outcomes 1:1 observation and crisis intervention showed a poor performance in the comparison of precision and recall without clinically meaningful combinations. Table 2 provides additional measures of classification performance for the remaining outcomes based on the potentially meaningful operational points.

**Table 2:** Perfomance Measures

	Sensitivity / Recall	Specificity	Positive Predictive Value / Precision	Negative Predictive Value	Prevalence	Detection Prevalence	Balanced Accuracy
Precision at least 20%							
Non-Response	1.00	0.00	0.23	1.00	0.23	1.00	0.50
Coercive Treatment	0.73	0.78	0.20	0.97	0.07	0.26	0.76
Long LOS	0.98	0.28	0.20	0.99	0.16	0.76	0.63
Short LOS	0.83	0.37	0.20	0.92	0.16	0.66	0.60
Precision at least 25%							
Non-Response	0.96	0.15	0.25	0.93	0.23	0.87	0.56
Coercive Treatment	0.48	0.89	0.25	0.96	0.07	0.13	0.69
Long LOS	0.94	0.48	0.25	0.98	0.16	0.58	0.71
Short LOS	0.61	0.65	0.25	0.90	0.16	0.39	0.63
Precision at least 33%							
Non-Response	0.52	0.69	0.33	0.83	0.23	0.36	0.61
Coercive Treatment	0.23	0.97	0.33	0.94	0.07	0.05	0.60
Long LOS	0.49	0.82	0.33	0.90	0.16	0.23	0.65
Short LOS	0.41	0.84	0.33	0.88	0.16	0.20	0.62

Outcomes without clinically meaningful operational points are not shown (Crisis Intervention & 1:1 Observation). Actual precision could be more than minimum precision. *TP* True Positive, *FP* False Positive, *TN* True negative, *FN* False Negative, Sensitivity = TP/(TP+ FN), Specificity = TN/(TN + FP), Positive Predictive Value = TP/(TP + FP), Negative Predictive Value = TN/(TN + FN), Prevalence = (TP + FN)/(TP + FP + TN + FN), Detection Prevalence = (TP + FP)/(TP + FP + TN + FN), Balanced Accuracy = (Sensitivity+Specificity)/2

**Fig. 3:**
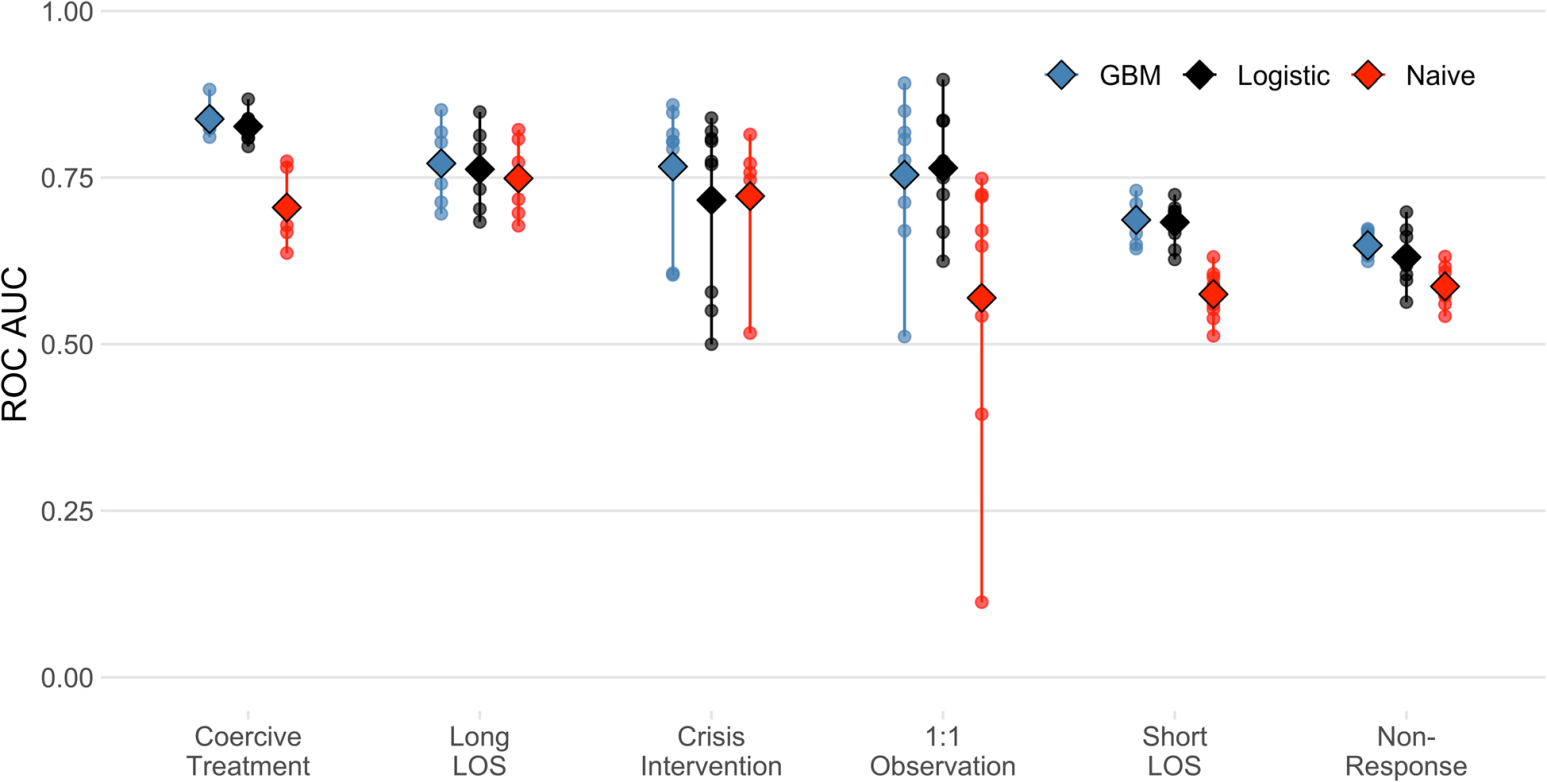
Performance in different study sites. One point represents one study site. The diamond represents the mean using the sites as units. ROC = Receiver operating characteristic. AUC = Area under the curve. LOS = Length of stay

As mentioned above, we trained each final model on patients discharged in 2017, leaving out one site in each training round, and evaluated each model’s predictive performance in patients discharged in 2018 only from the study site not included in the training. Figure 3 shows the differences in predictive performance measured by the area under the curve between the study sites. The models for coercive treatment, long LOS, short LOS and non-response to treatment showed relatively low variance in predictive performance. The models for crisis intervention and 1:1 observation performed very well in some study sites and very close to pure random classification, or worse, in others.

**Fig. 4:**
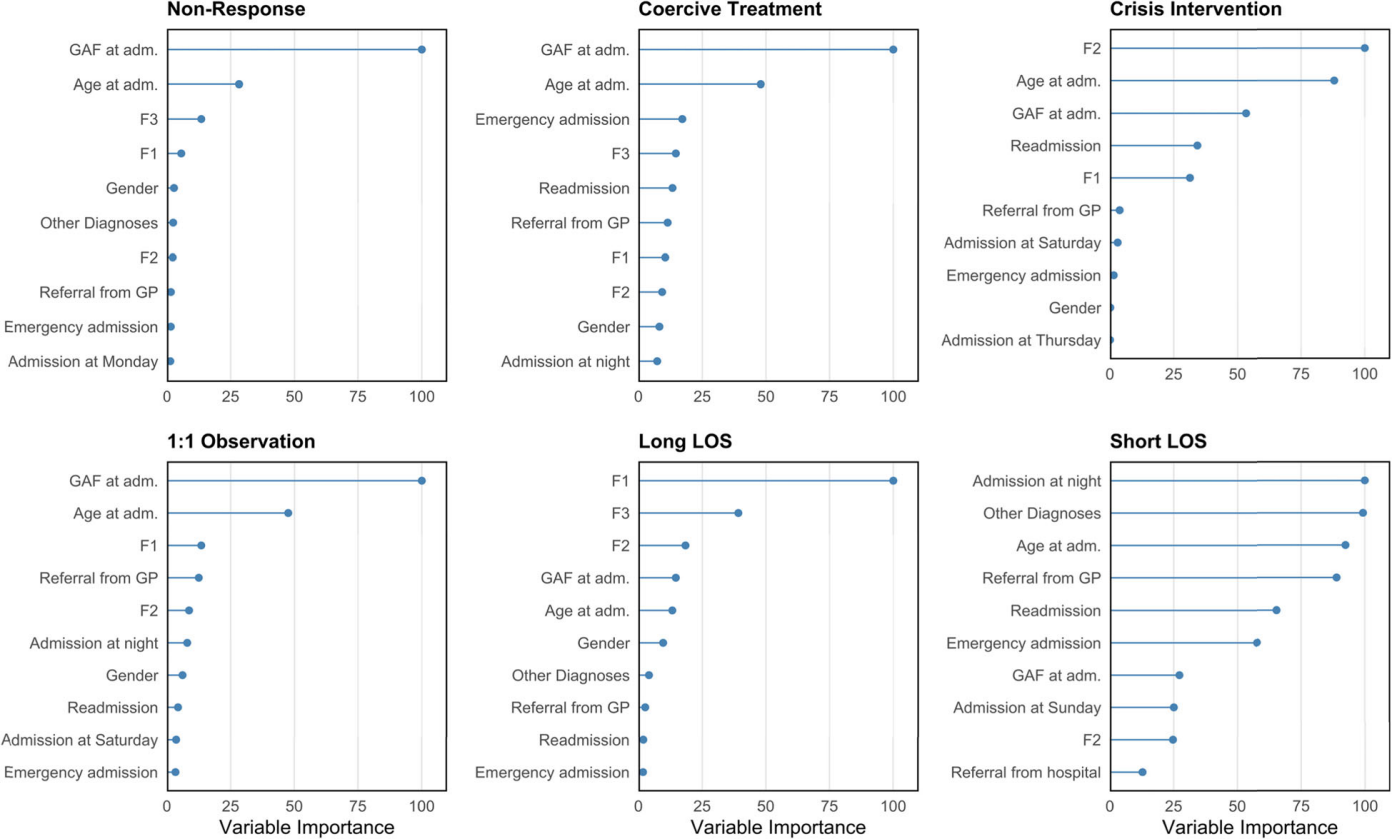
Importance of variables in predictions. F0/G3 = Organic mental disorders, F1 = Substance-related mental disorders, F2 = Schizophrenia, schizotypal and delusional disorders, F3 = Affective Disorders. GAF = Global Assessment of Functioning, Adm. = Admission, GP = General Practitioner

Figure 4 shows the top ten feature variables ordered by their importance in predicting the outcome variables in the GBM model. Variable importance is a dimensionless measure that represents the influence of each feature on the predictive performance relative to the other variables (the method is described in detail in 31). GAF at admission, age at admission and a basic diagnostic grouping at admission were important variables in most outcomes.

## Discussion

### Key findings

A common problem in the application of machine learning is availability of data at the point of decision making. The present study aimed at using routine data readily available at admission to predict aspects relevant to the organisation of psychiatric hospital care. A further aim was to compare the results of a machine learning approach to those obtained using a traditional method and those obtained using a naive baseline classifier.

The models’ performance, as measured by the area under the ROC, varied strongly between the predicted outcomes, with relatively high performance in the prediction of coercive treatment and 1:1 observations and relatively poor performance in the prediction of short LOS and non-response to treatment. The GBM performed slightly better than logistic regression. Both approaches were substantially better than a naive prediction based solely on basic diagnostic grouping.

The present results confirm previous studies suggesting inadequacy of the area under the ROC as a measure for predictive performance in unbalanced data, in our case data with many more negatives than positives [36]. The area under the ROC gave a misleadingly positive impression of the models for 1:1 observation and crisis intervention, while the precision and recall plots revealed a lack of sufficient precision for a clinically meaningful application.

Furthermore, we found relatively large differences in the areas under the ROCs between different hospital sites (Fig. 3). As described in the methods section, we trained each model in 8 hospital and used it to predict outcomes of patients from the left-out ninth hospital. Therefore, if the remaining ninth hospital had very different patients or provided care in a different way, the model would perform worse in this hospital than in the other hospitals that were more common to each other. This was probably the reason for the very low performance of the naive baseline classifier in predicting 1:1-observations in two study sites with areas under the curves below 0.5 (0.4 and 0.1, respectively), where the incidences of this event were very low (0.3 and 0.07%).

It is still unclear, which predictive performance is sufficient for beneficial application in routine clinical practice and this was out of the scope of the present study [37, 38]. Furthermore, different clinical applications might require their own trade-off decisions between reducing false alerts and increasing coverage of actual positives. We have chosen different configurations for comparison of model performance, borrowing from Tomašev et al. in their prediction of acute kidney injuries [13]. For instance, our GBM model for the prediction of coercive treatment, operationalised with a precision of at least 0.2 (see Table 2), gave a warning for 26% of all episodes at admission. Thereof, four false alerts were caused for each true alert and warnings were given in advance for 73% of all actual positive cases. The same model could be operationalised at a precision of 0.25, which gave a warning for 13% of all episodes, resulting in three false alerts for each positive alert and a warning for 48% of all actual positive cases.

Just because we can predict future events does not mean we should [39]. Very few clinical prediction models have undergone formal impact analysis, i.e. studying the impact of using the predictions on patient outcomes [40]. Patients’ benefit depends on how predictions are translated into effective decision making [41]. Predictions must be reasonably included in the clinical processes to create an actual benefit from better informed decisions. This requires a range of steps at the individual hospital level, such as integration into current IT, human resources and financial investment systems.

Even a perfectly integrated model must be used responsibly in clinical practice, and the exact framework for such application is currently under a broad discussion [42–44]. For instance, caregivers have to be trained in using the provided results, patients‘access to care has to remain equitable, real-world performance must be constantly scrutinised and responsibilities in case of errors have to be clear. Furthermore, predictions must not become self-fulfilling. Instead, a warning at admission for coercive treatment could be used to intensify non-invasive care with the aim to avoid coercive approaches, for instance.

### Our study in comparison to previous research

Less distinct diagnostic concepts [6–8], less standardization of care [9] and a broader spectrum of acceptable therapeutic regimes [10] make the prediction of outcomes in psychiatry more complex than in other medical disciplines [3–5]. An infamous example for these difficulties was the failure of the Medicare DRG system for psychiatry due to the inability to predict length of stay and associated hospital costs [45, 46]. Recent studies have often used a broad range of feature variables in studies restricted to specific settings and patients. Leigthon et al. [47] predicted remission after 12 months in 79 patients with first episode of psychosis with a wide range of demographic, socioeconomic and psychometric feature variables and reached an area under the ROC of 0.65. Koutsouleris et al. [48] also investigated remission in first episode of psychosis and reached a sensitivity of 71%, a specificity of 72% and a precision of 93% in 108 unseen patients with their top ten demographic, socioeconomic and psychometric predictor variables. Lin et al. [49] tried to distinguish treatment responders from non-responders prior to antidepressant therapy in 455 patients with major depression. They used single nucleotide polymorphisms from genetic analyses and other clinical data and reached an area under the AUC of 0.82. Common traits of these studies were the restriction to specific patient groups and the relatively small sample sizes. Furthermore, they mainly used data that might not be available during routine patient admission.

### Strengths and weaknesses of our study

A strength of this study was the large sample size over two distinct years and at nine study sites. This allowed us to include a broad range of the present spectrum of psychiatric inpatients and to develop models that should be applicable in most hospitals. Furthermore, we were able to test our models in patients that were treated in another calendar year and a different hospital and thereby reduce information leakage. A further strength of the present study was the restrictive inclusion of only feature variables that should be available at admission in most hospitals. Therefore, it should be possible to implement the present models in many hospitals without additional documentation effort.

A potential weakness of our study was the retrospective use of administrative routine data which entails potential validity concerns. The validity of routine hospital data for health services research is a frequently discussed topic [50, 51], and studies found both low [52] and high validity of such data [53]. However, the development of models for application in routine clinical practice necessitated the use of routinely generated data including the inherent caveats. A further limitation was the lack of time stamps for the diagnostic groupings. Patients were grouped in one of five basic diagnostic groups at admission and these groupings remained stable during an episode. However, we were not able to entirely rule out that these groupings might have been changed during the stay by staff in rare cases. A further limitation was the restriction to hospitals from one large provider of inpatient psychiatric services in the region of Hesse, Germany, which raises the question whether the predictive performance of our models would remain stable if applied in psychiatric hospitals with different circumstances.

## Conclusion

The present study has shown that administrative routine data can be used to predict aspects relevant to the organisation of psychiatric hospital care. Such predictions could be applied to efficiently support hospital staff in their very own decision making and thereby increase quality of care. Future research should investigate the predictive performance that is necessary for a tool to be accepted by care givers and provide an effective assistance in the care process for the benefit of both staff and patients.

## Data Availability

The datasets generated and/or analysed during the current study are not publicly available due confidentiality. The corresponding author will provide the script used for the statistical analyses upon reasonable request.

## Abbreviations

AUC: Area under the curve
GAF: Global Assessment of Functioning
GBM: Gradient Boosting Machine
LOS: Length of stay
PR-Plots: Precision and Recall plots
ROC: Receiver Operating Characteristic curve
